# Synergy Between Beta-Lactams and Lipo-, Glyco-, and Lipoglycopeptides, Is Independent of the Seesaw Effect in Methicillin-Resistant *Staphylococcus aureus*

**DOI:** 10.3389/fmolb.2021.688357

**Published:** 2021-09-09

**Authors:** Rutan Zhang, Ismael A. Barreras Beltran, Nathaniel K. Ashford, Kelsi Penewit, Adam Waalkes, Elizabeth A. Holmes, Kelly M. Hines, Stephen J. Salipante, Libin Xu, Brian J. Werth

**Affiliations:** ^1^ Department of Medicinal Chemistry, School of Pharmacy, University of Washington, Seattle, WA, United States; ^2^ Department of Pharmacy, School of Pharmacy, University of Washington, Seattle, WA, United States; ^3^ Department of Laboratory Medicine and Pathology, School of Medicine, University of Washington, Seattle, WA, United States

**Keywords:** seesaw effect, beta-lactam antibacterials, lipidomic analysis, vancomycin, daptomycin, dalbavancin, synergy, membrane fluidity

## Abstract

Methicillin-resistant *S. aureus* (MRSA) are resistant to beta-lactams, but synergistic activity between beta-lactams and glycopeptides/lipopeptides is common. Many have attributed this synergy to the beta-lactam-glycopeptide seesaw effect; however, this association has not been rigorously tested. The objective of this study was to determine whether the seesaw effect is necessary for synergy and to measure the impact of beta-lactam exposure on lipid metabolism. We selected for three isogenic strains with reduced susceptibility to vancomycin, daptomycin, and dalbavancin by serial passaging the MRSA strain N315. We used whole genome sequencing to identify genetic variants that emerged and tested for synergy between vancomycin, daptomycin, or dalbavancin in combination with 6 beta-lactams with variable affinity for staphylococcal penicillin binding proteins (PBPs), including nafcillin, meropenem, ceftriaxone, ceftaroline, cephalexin, and cefoxitin, using time-kills. We observed that the seesaw effect with each beta-lactam was variable and the emergence of the seesaw effect for a particular beta-lactam was not necessary for synergy between that beta-lactam and vancomycin, daptomycin, or dalbavancin. Synergy was more commonly observed with vancomycin and daptomycin based combinations than dalbavancin in time-kills. Among the beta-lactams, cefoxitin and nafcillin were the most likely to exhibit synergy using the concentrations tested, while cephalexin was the least likely to exhibit synergy. Synergy was more common among the resistant mutants than the parent strain. Interestingly N315-D1 and N315-DAL0.5 both had mutations in *vraTSR* and *walKR* despite their differences in the seesaw effect. Lipidomic analysis of all strains exposed to individual beta-lactams at subinhibitory concentrations suggested that in general, the abundance of cardiolipins (CLs) and most free fatty acids (FFAs) positively correlated with the presence of synergistic effects while abundance of phosphatidylglycerols (PGs) and lysylPGs mostly negatively correlated with synergistic effects. In conclusion, the beta-lactam-glycopeptide seesaw effect and beta-lactam-glycopeptide synergy are distinct phenomena. This suggests that the emergence of the seesaw effect may not have clinical importance in terms of predicting synergy. Further work is warranted to characterize strains that don’t exhibit beta-lactam synergy to identify which strains should be targeted with combination therapy and which ones cannot and to further investigate the potential role of CLs in mediating synergy.

## Introduction

All MRSA are resistant to traditional beta-lactams, but when combined with glycopeptides, lipopeptides, or lipoglycopeptides, synergistic antimicrobial activity is commonly observed, especially among strains with reduced susceptibility to the latter antimicrobial classes (Mehta et al., 2012; Werth et al., 2013b; Xhemali et al., 2018). Some investigators have attributed this synergy to the “seesaw effect” (Renzoni et al., 2017; Molina et al., 2020), a phenomenon where the susceptibility to beta-lactams increases with declining vancomycin or daptomycin susceptibility (Sieradzki and Tomasz, 1997; Ortwine et al., 2013). While it is intuitive that the seesaw effect between beta-lactams and glyco-, lipo-, and lipoglycopeptides could be related to synergy between these agents, this association has not been rigorously tested. While the seesaw effect is assumed to be a common feature of strains with reduced susceptibility to vancomycin or daptomycin, to measure it requires the comparison between a parent strain that is initially susceptible to vancomycin or daptomycin and isogenic strains that develop reduced susceptibility. There are a few well-characterized examples of such clinical strain pairs, but this is not something that can be easily determined through routine monitoring by clinical laboratories (Vignaroli et al., 2011; Werth et al., 2013a). Not all MRSA with reduced susceptibility to vancomycin (*i.e*., vancomycin intermediate *S. aureus*; VISA) or daptomycin exhibit the seesaw effect, and for most such clinical isolates, the emergence of the seesaw effect cannot be assessed due to absence of the parent strain.

In a previous study, we selected for a series of isogenic mutants by serial passage and identified isolates that do and do not exhibit the seesaw effect with a panel of six different beta-lactams (Hines et al., 2020). We found that lipidomic characteristics seem to correlate with the emergence or absence of the seesaw effect. In this study, we selected for a series of isogenic mutants against vancomycin, dalbavancin, or daptomycin using the well characterized MRSA strain N315, and evaluated the occurrence of seesaw effect and synergy with six different beta-lactams. The objective of this study was to determine whether the seesaw effect was necessary for synergy, to examine the changes in the lipidomic profiles after exposure to various beta-lactams, and to evaluate whether the lipidomic changes correlate with the occurrence of synergy with vancomycin, daptomycin, or dalbavancin.

## Materials and Methods

### Media, Antimicrobials and Strains

Serial passage, susceptibility testing, and time–kill experiments were performed in Mueller–Hinton II broth (MHB). Tryptic soy agar (TSA) was used for subculture of organisms and colony enumeration. Beta-lactams and vancomycin were purchased commercially from Sigma-Aldrich and Thermo Fisher Scientific. Ceftaroline-2-HCl and dalbavancin were acquired from Allergan, and daptomycin was purchased from Merck. The well-characterized MRSA strain N315 and three strains derived from N315 were evaluated. These strains were selected for by serial passage in escalating concentrations of vancomycin (VAN), leading to N315-VAN8, daptomycin (DAP), leading to N315-DAP1, and dalbavancin (DAL), leading to N315-DAL0.5, as described previously (Silverman et al., 2001; Hines et al., 2020).

### Reagents

LC/MS grade water and acetonitrile, ammonium acetate and brain heart infusion (BHI) media were purchased from Thermo Fisher Scientific. Phosphatidylcholines (PC) and phosphatidylethanolamines (PE) Standards for lipidomics were purchased from Avanti Polar Lipids and Nu-Chek prep and prepared as described previously (Hines K. M. et al., 2017; Hines KM. et al., 2017).

### Susceptibility Testing and Time–Kills

MICs were determined by broth microdilution at an inoculum of 10^6^ cfu/ml in accordance with CLSI guidelines (CLSI, 2017). MICs of vancomycin, dalbavancin, and daptomycin were also determined in the presence of 0.5x MIC (Table 1) of individual beta-lactams to assess the MIC lowering effects of subinhibitory beta-lactam concentrations. Time–kill experiments were performed in duplicate as previously described (Werth et al., 2013b; Werth, 2017). Briefly, 2 ml of MHB was inoculated with10^6^ cfu/mL of study organism and exposed to 0.5x MIC for both single and combination drug exposures. The average free peak plasma concentration for a given drug was used instead if 0.5x the MIC was greater than this value in order to avoid over-estimating clinically relevant effects as has been done previously (Werth et al., 2015; Werth, 2017). Exposures tested are summarized in Table 2 and included vancomycin, daptomycin, and dalbavancin each combined with nafcillin (NAF), meropenem (MEM), ceftriaxone (CRO), ceftaroline (CPT), cephalexin (LEX), and cefoxitin (FOX). Samples of 100 µL were taken at 0, 4, 8, and 24 h, diluted in sterile saline, and spiral plated for colony enumeration after 24 h incubation. Synergy was defined as a ≥2-log_10_cfu/mL reduction of the combination over the most active single agent, antagonism was defined as ≥1-log_10_cfu/mL growth compared with the most active single agent, and other interactions were considered indifferent.

**TABLE 1:** Strains used in this study and their antibiotic susceptibility and genetic profiles. The seesaw effect was most pronounced with cephalexin (LEX) and nafcillin (NAF) across the 3 mutant strains. Vancomycin (VAN), daptomycin (DAP), dalbavancin (DAL), nafcillin (NAF), cephalexin (LEX), meropenem (MEM), ceftriaxone (CRO), cefoxitin (FOX), ceftaroline (CPT).

				Cross-resistance MIC (µg/ml)								Genetic variants	
Strain name	Selection drug	VAN	DAP	DAL	NAF	LEX	MEM	CRO	FOX	CPT	Gene Name	Nucleotide Change	Predicted Amino Acid Change
N315	−	0.5	0.125	0.0039	16	32	16	512	128	1	−	−	−
N315-Dap1	DAP	1	1	0.0039	0.25	0.125	32	512	128	0.5	*walK*	784 A → G	Lys262Glu
											*yjbH*	257 G → A	Gly86Asp
											*vraS*	353 T → C	Leu118Ser
N315-Dal0.5	DAL	4	1	0.5	>32	8	16	2048	128	0.5	*walK*	1278 G → A	Met426Ile
											*vraT*	20 C → T	Ser7Leu
											*SA1741*	242 C → A	Ala81Asp
N315-Van8	VAN	8	1	1	0.5	1	8–16	64	64–128	0.25	*asp1*	841 A → G	Met281Val
											*norA*	737 G → C	Gly246Ala

**TABLE 2:** Increase in killing activity (−log_10_cfu/mL) of the combinations compared to the most active single agents from the time kills. Synergy (green) is ≤ −2 log_10_cfu/mL; −2–1 log_10_cfu/mL is improved activity (yellow), −1−0 log_10_cfu/mL is no interaction (red).

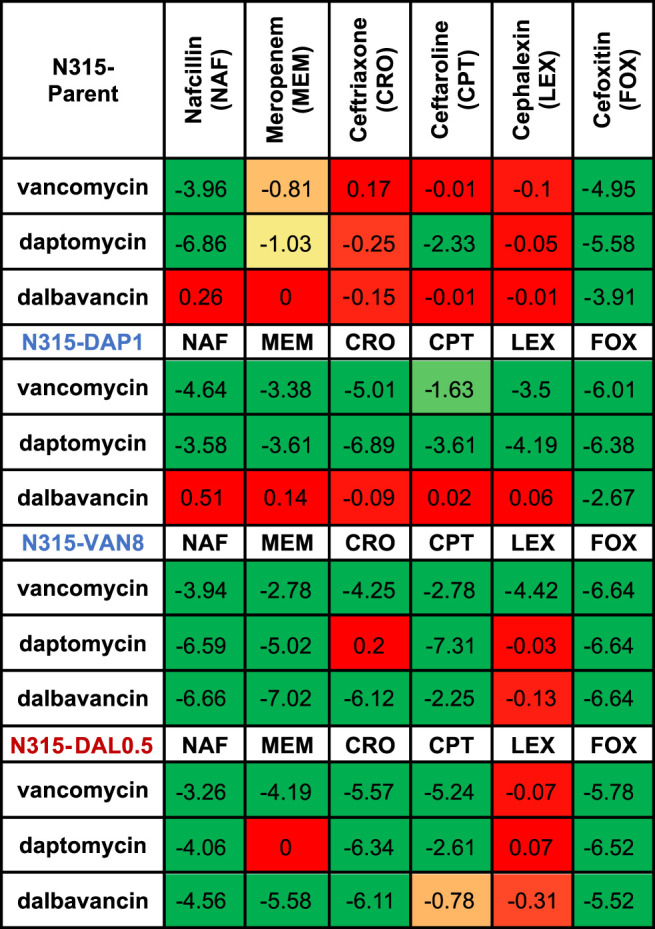

### Whole Genome Sequencing

DNA from N315 and antibiotic-selected derivatives was extracted using the Ultraclean microbial DNA isolation kit (Mo Bio). Sequencing libraries were prepared as described elsewhere (Roach et al., 2015; Salipante et al., 2015), with sequencing performed using an Illumina MiSeq (Illumina, San Diego, CA, United States) with 150-bp paired-end chemistries. Sequence analysis was performed to identify single nucleotide mutations and insertion and deletion mutations in coding sequences as previously (Jorth et al., 2017), against the reference genome of N315 (GenBank Accession BA000018). Sequence variants were annotated using SnpEFF (Cingolani et al., 2012). Whole genome sequencing data from this study are available from the NCBI Sequence Read Archive (SRA; http://www.ncbi.nlm.nih.gov/sra) under BioProject number PRJNA547605.

### Preparation of N315 and N315 Mutants for Lipidomics

The N315 parent strain and three N315 mutants (N315-DAP1, N315-VAN8 and N315-DAL0.5) were grown in 1 ml of BHI medium with addition of various beta-lactams targeting at different penicillin binding proteins (PBPs), including nafcillin (PBP non-specific), cephalexin (PBP3), meropenem (PBP1), ceftriaxone (PBP2), cefoxitin (PBP4), and ceftaroline (PBP2a) as shown in Table 1. Each strain was grown in the presence and absence of each beta-lactam in quadruplicate in subinhibitory concentrations equal to 0.5 time the MIC unless that value exceeded the maximum plasma concentration in which the average Cmax concertation was used instead.

### Lipid Extraction

Briefly, bacteria broth was collected after cultivation overnight in the absence or presence of individual beta-lactams, rinsed with 1x PBS, spun and dried with a speed-vac. 150 μL of water was then added to the pelleted and dried bacteria. The resulting suspensions were sonicated in an ice bath for 30 min to dislodge the dried pellets and homogenize the suspension. A chilled solution of chloroform and methanol (1:2 v/v, 600 μL) was added to each tube, followed by 5 min of vortex and the addition of 150 μL of chilled chloroform and 150 μL of chilled water. The samples were then rigorously vortexed for 1 min and centrifuged for 10 min at 4°C and 2,000 × *g* to separate the organic and aqueous layers. The organic layers were collected to clean 1.5 ml polypropylene microcentrifuge tubes (Fisher Scientific, Waltham, MA, United States) and dried in a vacuum concentrator. The dried lipid extracts were reconstituted with 500 µL of 2:1 acetonitrile/methanol and transferred to glass vials for storage prior to LC-MS analysis.

### Liquid Chromatography

Bacterial lipids were separated by a Waters UPLC (Waters Corp., Milford, MA, United States) as described previously (Hines K. M. et al., 2017; Hines KM. et al., 2017). Briefly, hydrophilic interaction liquid chromatography (HILIC) was performed with a Phenomenex Kinetex HILIC column (2.1 × 100 mm, 1.7 µm) maintained at 40°C at a flow rate of 0.5 ml/min. The solvent system consisted of: A) 50% acetonitrile/50% water with 5 mM ammonium acetate; and B) 95% acetonitrile/5% water with 5 mM ammonium acetate. The linear gradient was as follows: 0–1 min, 100% B; 4 min, 90% B; 7–8 min, 70% B; 9–12 min, 100% B. A sample injection volume of 5 µL was used for all analyses.

### Ion Mobility-Mass Spectrometry

The Waters Synapt G2-XS platform was used for lipidomics analysis. Effluent from the UPLC was introduced through the electrospray ionization (ESI) source. ESI capillary voltages of +2.0 and −2.0 kV were used for positive and negative analyses, respectively. Additional ESI conditions were as follows: sampling cone, 40 V; extraction cone, 80 V; source temperature, 150°C; desolvation temperature, 500°C; cone gas, 10 L/h; desolvation gas, 1000 L/h. Mass calibration over *m/z* 50-1200 was performed with sodium formate. Calibration of ion mobility (IM) measurements was performed as previously described (Hines et al., 2016). IM separation was performed with a traveling wave height of 40 V and velocity of 500 m/s. Data was acquired for *m/z* 50-1200 with a 1 s scan time. Untargeted MS/MS (MS^E^) was performed in the transfer region with a collision energy ramp of 35–45 eV. Mass and drift time correction was performed post-acquisition using the leucine enkephalin lockspray signal.

### Cell Membrane Fluidity Assay

The effects of beta-lactam exposure on membrane fluidity was measured in each of the strains as previously described.(Lew et al., 2021). Briefly, the N315 parent strain and N315 mutants were grown overnight in 5 ml of BHI medium in the presence and absence of each of the beta-lactams tested in the time-kills at subinhibitory concentrations at 37°C, pelleted, and then resuspended into saline to a 0.9 McFarland suspension. CM fluidity was subsequently measured by polarizing spectrofluorometery using Synergy H1 Hybrid Multi-Mode Reader (BioTek Instrument, Inc., Winooski, VT, United States) with 1,6-diphenyl-1,3,5-hexatriene (DPH) as the probe. The detailed methods for calculating fluorescence polarization (FP) have been described previously (Lentz, 1989; Mishra et al., 2021). There is an inverse relationship between FP values and CM fluidity (Mishra et al., 2021). Each FP value was obtained based on 8 technical replicates.

### Data Analysis

Data alignment, chromatographic peaks detection, and normalization were performed in Progenesis QI (Nonlinear Dynamics). A pooled quality control sample was used as the alignment reference. The default “All Compounds” method of normalization was used to correct for variation in the total ion current amongst samples. PCA analysis was performed with the online tool, MetaboAnalyst 4.0 (Chong et al., 2018). Pearson correlation coefficients were obtained using the Correlation function in the Analysis Toolpak add-in in Excel and visualized by an R package, ComplexHeatmap (Gu et al., 2016). Lipid profile heatmap was generated using ClustVis (Metsalu and Vilo, 2015). Student’s t-tests for two groups were performed using a two-tailed distribution and equal variance. Lipid identifications were made based on *m/z* (within 10 ppm mass accuracy), retention time, and CCS with an in-house version of LipidPioneer, modified to contain the major lipid species observed in *S. aureus*, including free fatty acids (FFAs), DGDGs, PGs, CLs, and LysylPGs with fatty acyl compositions ranging from 25:0 to 38:0 (total carbons: total degree unsaturation), and LiPydomics (Hines KM. et al., 2017; Ulmer et al., 2017; Ross et al., 2020).

## Results

### Susceptibility Testing and Time–Kills

Susceptibility of each of the N315-derived strains are listed in Table 1. As shown in the table, N315-DAP1 exhibited strong seesaw effect with cephalexin and nafcillin, with 8 and 6-log_2_ fold-change in MIC, respectively, compared to the N315 parent strain. N315-Van8 strain, aside from the above 2 beta-lactams inducing 5 log_2_ fold-change, ceftriaxone also showed significant decreases in MIC with 3 log_2_ fold-change relative to the parent. For N315-Dal0.5 strain, no general seesaw effect was observed in our susceptibility testing, notably, ceftriaxone and nafcillin even displayed increased MICs. Generally there was cross-resistance among VAN/DAL/DAP similar to what has been reported previously (Hines et al., 2020).

Time-kills results are summarized in Table 2 and detailed kill curves are illustrated in Supplementary Figures S1–S6. Synergistic sensitivity to drug combinations of 3 peptide-based antimicrobials and 6 beta-lactams was commonly observed among all three N315-derived mutants with the exceptions that the combination of dalbavancin and 5 beta-lactams did not exhibit synergistic killing against N315-DAP1 and most combinations with cephalexin did not display synergy. In contrast, synergy was less common for the N315 parent strain. Interestingly, we found that the seesaw effect was not necessary for synergy between beta-lactams and VAN/DAP/DAL against MRSA. Although N315-DAL0.5 did not exhibit the seesaw effect (Table 1), this strain displayed general synergistic effect for most combinations of VAN, DAP or DAL with beta-lactams (Table 2); for example, with increases in killing activity up to 5.78, 6.52 and 6.11 log_10_CFU/mL, for drug combinations of vancomycin and cefoxitin, daptomycin and cefoxitin, as well as dalbavancin and ceftriaxone, respectively. It is also noteworthy that among all tested beta-lactams, cefoxitin and nafcillin were the most likely to exhibit synergistic activity, while cephalexin was the least likely to exhibit synergy.

Changes to the MICs of VAN, DAP, and DAL in the presence of sub-inhibitory concentration of individual beta-lactams relative to the MICs of the peptide-based drugs alone were also evaluated (Table 3). As shown in the table, most of the MICs of the three drugs significantly decreased in the presence of beta-lactams with the exception of cephalexin, indicating wide occurrence of synergy.

**TABLE 3:** Decrease in MIC (1/(fold change) of the combinations compared to the most active single agents from the susceptibility tests. Synergy (green) is 0.25 MIC; 0.25–0.5 MIC is improved activity (yellow), 0.5–1 MIC is no interaction (red).

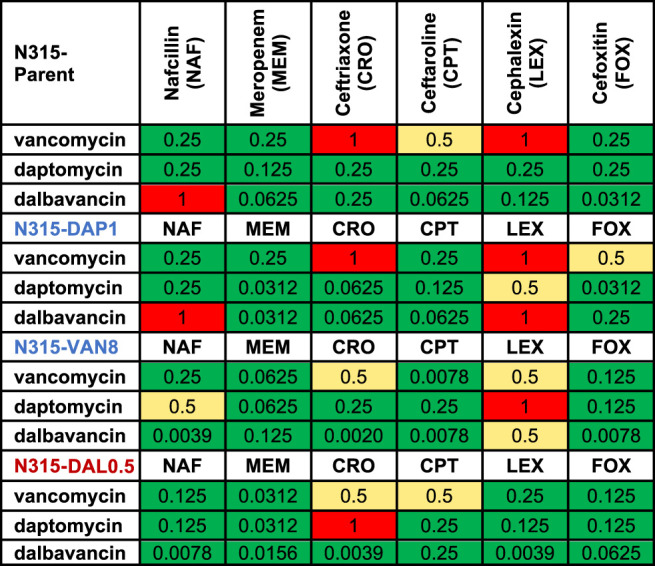

### Whole Genome Sequencing

Whole genome sequencing (WGS) was performed on all selected strains to identify mutations which arose relative to the parental N315 (Table 1). Mutations affecting the *vraTSR* operon and the histidine kinase, *walK* were detected in N315-DAP1 and N315-DAL0.5 but not in N315-VAN8. Alternatively, N315-VAN8 acquired a variant in *asp1,* encoding accessory secretory system protein, and *norA*, a known contributor to quinolone resistance, neither of which has been clearly implicated in the emergence of VAN/DAL/DAP resistance previously.

### Lipidomic Analysis of Beta-Lactam Exposed Strains

**FIGURE 1:**
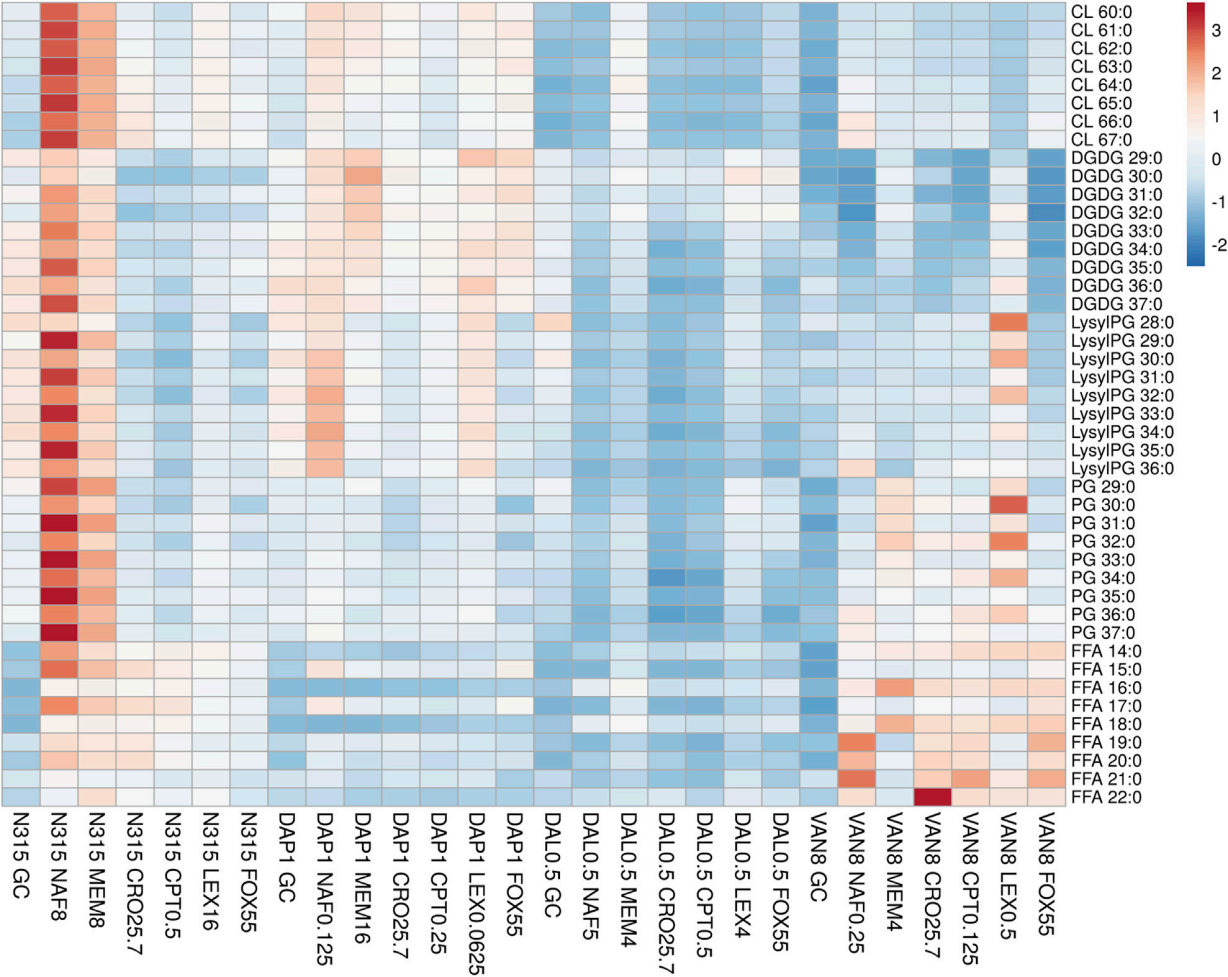
The distribution of main lipids in N315 mutants (DAL0.5, DAP1 and VAN8) and the N315 parent strain treated with six beta-lactams. CPT, ceftaroline; CRO, ceftriaxone; FOX, cefoxitin; LEX, cephalexin; MEM, meropenem; NAF, nafcillin; GC, drug-free growth control.

DAP-, VAN- and DAL-non-susceptible N315 mutants (4 replicates) as well as the N315 parent strains (4 replicates) were treated with 6 beta-lactams targeting different PBPs at the same concentrations used in the time kill experiments and combination MICs (see Experimental Section), then harvested for lipidomic analysis (Figure 1; NAF, MEM, CRO, CPT, LEX, FOX). Major lipids were identified as shown in Supplementary Table S1. PCA analysis of the lipidome in negative mode (Supplementary Figure S7) showed clear clustering of biological replicates, indicating good reproducibility of the sample preparation process and the LC-MS analysis of lipids. As shown in the PCA plot, the cluster of N315-VAN8 strains overlapped with the cluster of the N315 parent strains, indicating that they have similar lipidomic phenotype after being treated with beta-lactams. The heatmap intuitively shows the variation of major lipids observed in N315 mutant and parent strains treated with beta-lactams (Figure 1). Specifically, most of the cardiolipins (CLs) in N315-DAP1 strains and N315 strains were upregulated after being treated with beta-lactams, relative to the growth control (GC) strain without beta-lactam treatment. In N315-VAN8 samples treated with beta-lactams, most of the FFAs and PGs were significantly elevated, compared to VAN8 GC and N315 GC. For the N315 parent strain treated with nafcillin or meropenem, nearly all classes of lipids were highly expressed. For N315-Dal0.5, the presence of beta-lactams has only minor effect on the lipid profiles, in comparison with the GC strain.

### Correlation of Lipid Levels and MICs

**FIGURE 2:**
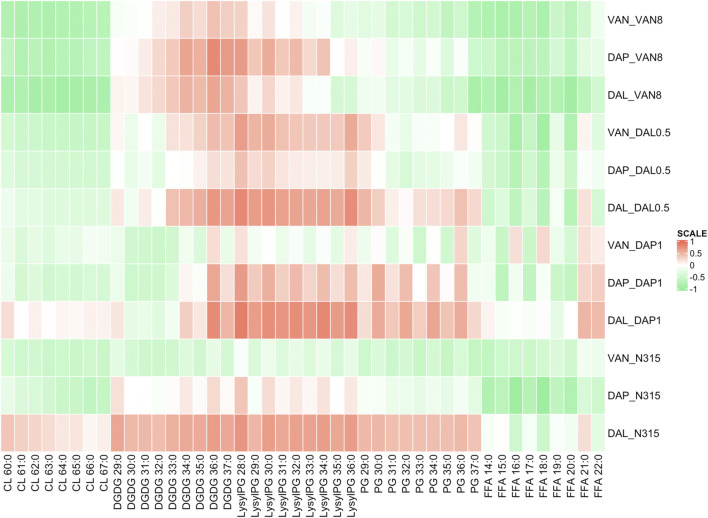
Pearson correlation analysis showing the difference in individual lipid species abundance in a strain given a series of beta-lactam exposure vs the change in VAN, DAL, or DAP MIC given the same beta-lactam exposure. Green indicates that as an individual lipid increases, the MIC of VAN/DAP/DAL in the presence of beta-lactam decreases, while red indicates the opposite.

Using Pearson correlation analysis, the difference in individual lipid species abundance in a strain given a series of beta-lactam exposure was correlated with the changes in VAN, DAL, or DAP MIC for the same strain given the same beta-lactam exposure (Figure 2). In general, the MIC changes for the combination of VAN/DAP/DAL and beta-lactams had negative correlations with the levels of CLs, except for the N315-DAP1 strain and N315 parent strain treated with dalbavancin with or without beta-lactams. Conversely, the MICs changes for VAN/DAP/DAL in each strain were positively correlated with LysylPGs, except for the N315-DAP1 strain and N315 parent strain treated with vancomycin. For FFAs, there was an overall negative correlation with the MIC changes, and the correlation seemed to vary with fatty acid chain length. For DGDGs and PGs, there was no consistent trend observed for the correlation between the MIC changes and lipid levels among all four strains.

### Correlation of Lipid Levels and Time-Kills

**FIGURE 3:**
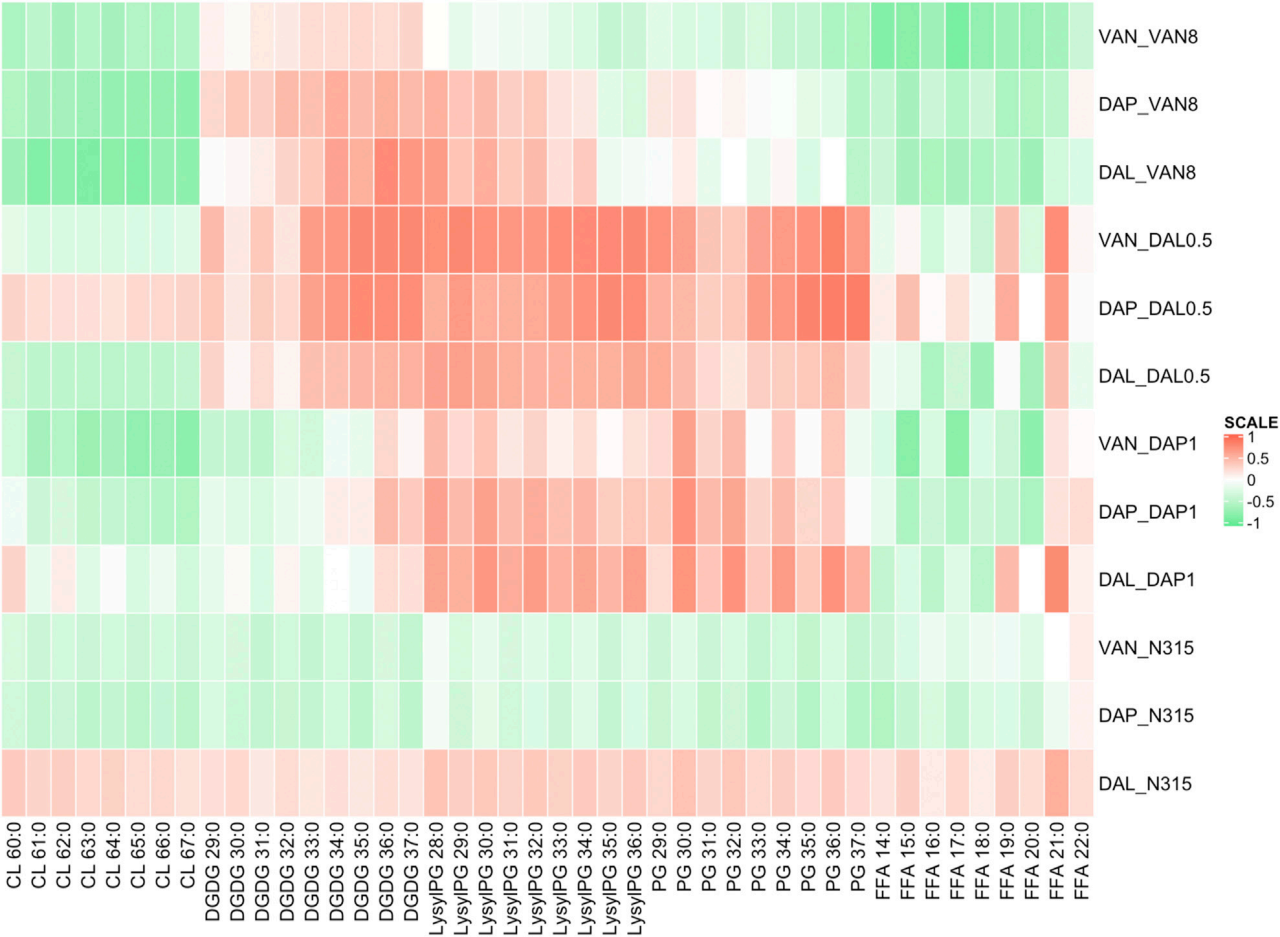
Pearson correlation analysis showing the difference in individual lipid species abundance given a beta-lactam exposure vs the change in log_10_CFU/mL of the combination treatments of VAN, DAL, or DAP given the same beta-lactam exposure in time kills. Green indicates that as an individual lipid increases, the bacterial survival synergistically declined with beta-lactam exposure, while red indicates the opposite.

The difference in individual lipid species abundance for a strain given a series of beta-lactam exposure was also correlated with the changes in activity in time kills between VAN, DAL, or DAP alone or in combination with that same beta-lactam exposure using Pearson correlation analysis (Figure 3). For CLs, the correlation between lipid levels and time-kill changes showed similar trend as between lipid levels and MICs. The levels of LysylPGs and PGs were mainly positively correlated with the changes in time-kills of combo treatments relative to single VAN/DAP/DAL treatment, except for the N315-VAN8 strain treated with vancomycin and the N315 parent strain treated with vancomycin and daptomycin. For FFA, there was an overall negative correlation with the presence of synergy, and consistent with the MIC related correlations, such correlation seems to vary with fatty acid chain length. However, the correlation between synergies and the levels of DGDGs varied for specific lipid species and for the strains examined.

## Discussion

In this study, we demonstrated that synergy between VAN/DAL/DAP and beta-lactams against MRSA is not dependent on the emergence of the seesaw effect. While N315-DAL0.5 did not exhibit the beta-lactam seesaw effect after becoming resistant to these peptide drugs, most combinations of VAN, DAP, or DAL with beta-lactams were still synergistic against this strain. Despite that N315-DAL0.5 and N315-DAP1 both acquired mutations in the *vraTSR* operon and *walK*, the seesaw effect phenotype was discordant. We do not know how the function of these genes was affected by the different mutations, but this discordance suggests that WalKR and/or VraTSR modulate cell envelope metabolism that favors or hinders beta-lactam susceptibility independently from any effect on VAN/DAP/DAL susceptibility. However, we also report the emergence of the seesaw effect and VAN/DAP/DAL cross resistance in a VISA strain that did not carry mutations in genes known to affect susceptibility these drugs or cell envelope metabolism. This finding reinforces the idea that beta-lactam susceptibility and VAN/DAP/DAL susceptibility are indeed closely linked metabolically.

Another pivotal finding in this research is that cefoxitin was among the least likely of the beta-lactams to show any seesaw effect but was consistently synergistic with VAN/DAL/DAP. Conversely, all strains, even N315-DAL0.5, exhibited some degree of the seesaw effect with cephalexin, but cephalexin was rarely synergistic with VAN/DAL/DAP at the concentrations tested. The PBP-nonselective nafcillin frequently exhibited the seesaw effect but also commonly exhibited synergy with VAN/DAL/DAP. Dalbavancin was less likely than vancomycin or daptomycin to be synergistic with companion agents overall, but specifically it was less likely to exhibit synergy against the parent strain and the N315-DAP1 strain, which both had very low dalbavancin MIC values. Due to these low MICs it remains possible that the time kills underestimated the synergistic effects against these strains and that greater enhancement would be seen at therapeutic concentrations. However other studies have shown only modest improvement of dalbavancin in combination with ceftaroline in *in vitro* PK/PD models against dalbavancin susceptible strains (Kebriaei et al., 2019). Alternatively, synergy may be more common or more pronounced against strains with reduced susceptibility to dalbavancin. Additionally, the synergistic effects of these drug combinations were only observed in mutants from a single genetic background and only in *in vitro* time kills, so these data should not be used to select optimally synergistic combinations in the clinical setting.

Although many researchers have shown evidence that LysylPGs are increased in daptomycin-resistant MRSA, our lipidomic study (Figure 1), clearly showed that the expression of LysylPGs could be altered by treatment with different types of beta-lactams. The reduction in LysylPGs observed when N315-DAP1 was treated with CRO or FOX, relative to the growth control, DAP1 GC (Figure 1 and Supplementary Figure S8), suggests that beta-lactam exposure reversed the changes in LysylPGs associated with the emergence of resistance. Furthermore, we found increases in the negatively charged CLs in most of the N315-DAP1 samples treated with beta-lactams (Figure 1 and Supplementary Figure S8). Moreover, CLs were also highly expressed in the other beta-lactam exposed N315 derivatives compared to their respective non-treatment growth controls (Figure 1 and Supplementary Figures S10–S12), indicating that beta-lactams appear to upregulate the synthesis of CLs. As shown in the Figure 1, it is also noteworthy that different PBP-targeting beta-lactams have different impact on the composition of cell membrane lipids for each strain derived from the common N315 ancestry.

For the three N315 mutants, synergistic activity, measured by either combination MIC or time kill, mostly (with two exceptions) positively correlated with the levels of LysylPGs. LysylPGs contain a positively charged headgroup, and increased level of LysylPGs has been associated with daptomycin resistance (Peleg et al., 2012; Jiang et al., 2019). Since LysylPGs are synthesized by lysinylation of negatively charged PGs by MprF (Peschel et al., 2001; Oku et al., 2004), the ratio of PGs/LPGs is a key indicator of charge changes. As expected, for all N315 mutants, the changes of PGs/LPGs are positively correlated with the synergies (negatively correlated with the colony counts) between DAP and beta-lactams (Supplementary Figure S13). However, whether such increases in positive charge could also affect the binding of VAN or DAL is unknown. In contrast, the expression of CLs in most of the N315 mutants generally displayed negative correlation with synergistic reductions in survival or MICs, which might be related with the remodeling of cell membrane (Khan et al., 2019). Increased CL content has been found to increase fluidity and decrease mechanical stability of the lipid bilayer (Unsay et al., 2013). Mutations or downregulation in *cls* have also been associated with daptomycin resistance (Camargo et al., 2008; Peleg et al., 2012; Jiang et al., 2019; Khan et al., 2019). Interestingly, daptomycin was shown to attract fluid lipids in the membrane, causing delocalization of cell-wall synthesis-related proteins, proton leakage, and eventual death (Muller et al., 2016). For most of the N315 mutants, there was no clear correlation between DGDGs, PGs and changes in VAN/DAL/DAP MICs. However, we note that MIC synergies do not necessarily correlate with time-kill synergies as PGs showed overall positively correlated with time-kill synergies (similar to LysylPGs). As for FFAs, except some fatty acids with longer acyl chains, an overall negative correlation with changes in MIC/time-kill synergies was observed for most of the N315 mutants. It has been reported that a higher proportion of branched-chain fatty acids are associated with higher susceptibility to daptomycin (Boudjemaa et al., 2018). However, our lipidomic method does not allow the determination of straight or branched-chain fatty acids at this time, so future work is needed to validate this hypothesis.

In previous studies, beta-lactam exposure in daptomycin-resistant MRSA leads primarily to decreased cell membrane (CM) fluidity, but sometimes it is increased or unchanged (Lew et al., 2021; Mishra et al., 2021). Thus, different beta-lactam exposures and different mutations may have distinct impacts on the CM fluidity as a result of various lipids perturbations. The DPH based fluorescence polarity assay (Supplementary Figure S14) showed there were no general changes in CM fluidity for the majority of N315 mutants exposed to beta-lactams, which generally showed synergistic activity, indicating CM fluidity may not be the key factor in facilitating the general beta-lactam synergy. Correlation analysis between CM fluidity and synergistic killing (Supplementary Figure S15) did not display consistent trends across the strains, suggesting that alteration of CM fluidity by lipids is confined to the strain level.

While MRSA elaborates 5 PBP subtypes, the seesaw effect between beta-lactams and peptide antibiotics, including daptomycin and vancomycin, usually stems from the vulnerability of PBP2a due to its dependence on functional membrane microdomains, wall teichoic acids, and the *PrsA* chaperone (Jousselin et al., 2015; Renzoni et al., 2017). Interference with any of these elements impairs PBP2a function and renders MRSA susceptible to beta-lactams (Foster, 2019). Both *walK* and *vraTSR* mutations are commonly observed in MRSA and their regulation of cell wall synthesis is well documented (Roch et al., 2017). It is likely that disruptions in cell membrane metabolism mediated by mutations in these genes could sensitize MRSA to beta-lactams by interfering with PBP2a trafficking. It’s also noteworthy that the observed single nucleotide polymorphisms (SNPs) within *walK* and *vraTSR* may act as hot spots for gain-in-function phenotypes of synergies, similar with the association between SNPs within the *mprF* open reading frame and acquisition of daptomycin resistance (Bayer et al., 2015). Additionally, *yjbH* mutation observed in N315-DAP1 was also found to be associated with the acquisition of beta-lactam resistance by affecting PBP4 levels and peptidoglycan cross-linking, despite its main role in regulating oxidative burst by binding to the transcriptional regulator Spx and controlling its degradation via the proteasome-like ClpXP protease (Gohring et al., 2011; Engman et al., 2012). However, It’s also possible that the inactivation of proteolysis triggered by *yjbH* mutation may directly mediate changes of susceptibility to beta-lactams by pathways regulating cell wall metabolism (Baek et al., 2014). While PBP4 expression has been shown to be important for maintaining resistance to oxacillin and nafcillin among community acquired strains of MRSA, knocking out PBP4 in N315, a classic nosocomial strain of the United States 100 lineage, did not impact nafcillin susceptibility; therefore, the contribution of this mutation is unclear (Memmi et al., 2008).

Like other *in vitro* studies with a similar design, this study has some limitations. We analyzed a set of 4 isogenic strains, which allows for better comparison across genotypes but does not allow for universal claims to be made about the relationship between beta-lactam synergy and the beta-lactam seesaw effect. However, our conclusion that synergy between glyco-/lipo-/lipoglyco-peptides and beta-lactams is independent of the seesaw effect between these agents in MRSA is robust and was demonstrated with several beta-lactams and glyco-/lipo-/lipoglycopeptide combinations among our strains. Furthermore, we are confident that beta-lactams are likely synergistic with glyco-/lipo-/lipoglyco-peptides, in part, because of beta-lactam effects on lipid metabolism, which enhances the activity of these drugs. Another potential limitation is that we exposed each strain to beta-lactam concentrations equal to 0.5x the MIC in time kills and prior to lipid analysis. Since these concentrations varied from strain to strain, some strains were exposed to absolute beta-lactam concentrations over 10 times greater than other strains, which could underestimate effects of higher concentrations on strains exhibiting the seesaw effect. Conversely, by using similar relative exposures among strains, we avoided underestimating effects in strains that did not exhibit the seesaw effect. With these limitations and tradeoffs in mind, we caution against readers who may be tempted to extrapolate these findings to determine the optimal beta-lactam/peptide combination for clinical use.

Future studies are needed to understand the roles of *vraTSR* and *walKR* in manifesting the seesaw effect and to uncover the molecular mechanisms by which *norA* and/or *asp1* affect cell envelope metabolism. Additional work is also needed to unveil the underlying mechanism(s) by which beta-lactams modulate lipidomic phenotypes and how the lipidomic changes affect the synergies between VAN/DAP/DAL and beta-lactams in MRSA.

## Data Availability

The datasets presented in this study can be found in online repositories. The names of the repository/repositories and accession number(s) can be found below: http://www.ncbi.nlm.nih.gov/sra under BioProject number PRJNA547605.
