# Supplementary material for: Synergy Between Beta-Lactams and Lipo-, Glyco-, and Lipoglycopeptides, Is Independent of the Seesaw Effect in Methicillin-Resistant Staphylococcus aureus
Source: Front Mol Biosci. 2021 Sep 9;8:688357. doi: 10.3389/fmolb.2021.688357 (PMC8503943; doi:10.3389/fmolb.2021.688357)
Supplement: Supplementary file 1 [file DataSheet1.pdf]

Supplementary for Synergy between beta-lactams and lipo-, glyco-, and lipoglycopeptides, is independent of the seesaw effect in Methicillin-Resistant *Staphylococcus aureus*

Rutan Zhang[1], Ismael A. Barreras Beltran[2], Nathaniel K. Ashford[2], Kelsi Penewit[3], Adam Waalkes[3], Elizabeth A. Holmes[3], Kelly M. Hines[1#], Stephen J. Salipante[3], Libin Xu[1*], Brian J. Werth[2*]

1. Department of Medicinal Chemistry, School of Pharmacy, University of Washington, Seattle, WA, USA

2. Department of Pharmacy, School of Pharmacy, University of Washington, Seattle, WA, USA

3. Department of Laboratory Medicine, School of Pharmacy, University of Washington, Seattle, WA, USA

# current address: Department of Chemistry, University of Georgia, Athens, GA, 30602, USA

* Correspondence:
Libin Xu, PhD, libinxu@uw.edu
Brian J. Werth, PharmD, bwerth@uw.edu

Figure S1. Time kill synergy studies illustrating survival of each N315 strain over 24 hours with exposure to subinhibitory concentrations of nafcillin (NAF), dalbavancin (DAL), vancomycin (VAN), daptomycin (DAP) alone and in combination or drug free growth controls (GC).

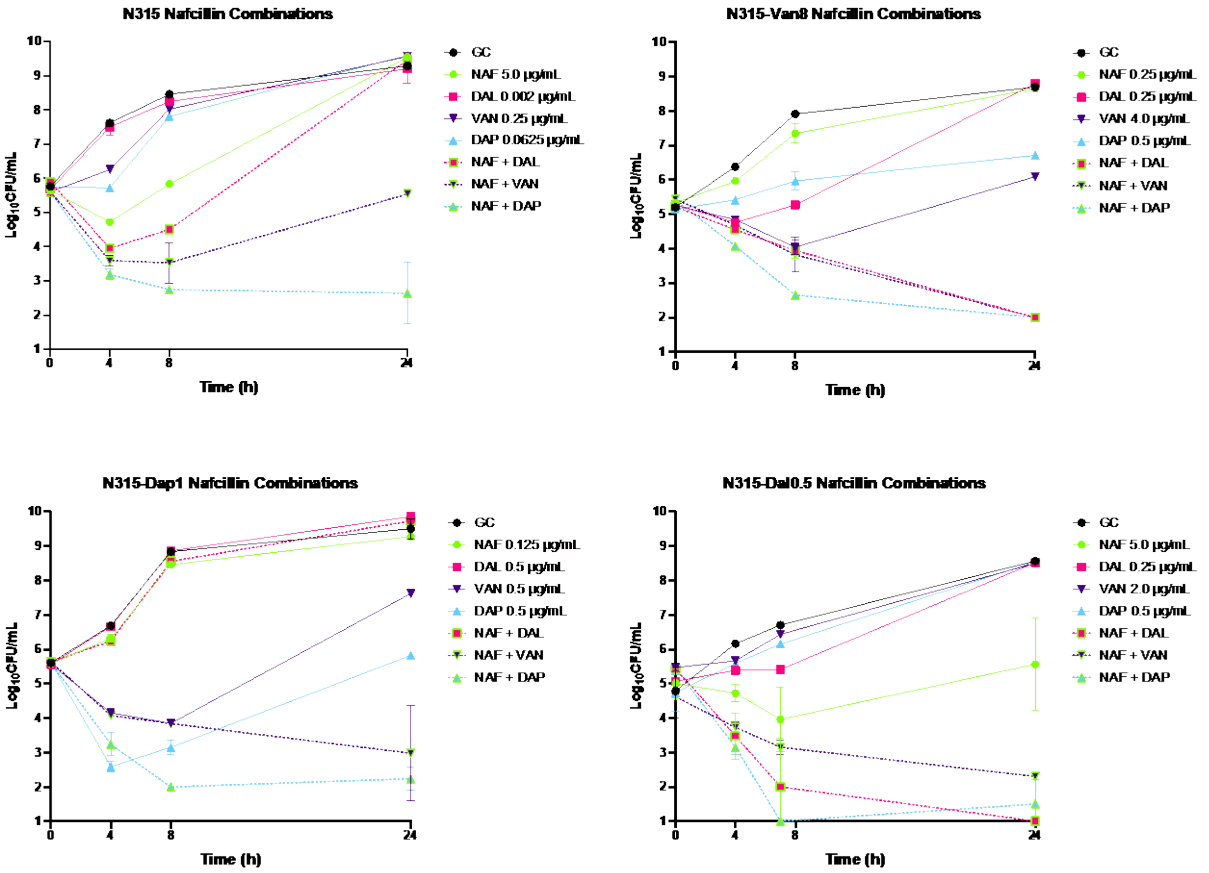

Figure S2. Time kill synergy studies illustrating survival of each N315 strain over 24 hours with exposure to subinhibitory concentrations of meropenem (MEM), dalbavancin (DAL), vancomycin (VAN), daptomycin (DAP) alone and in combination or drug free growth controls (GC).

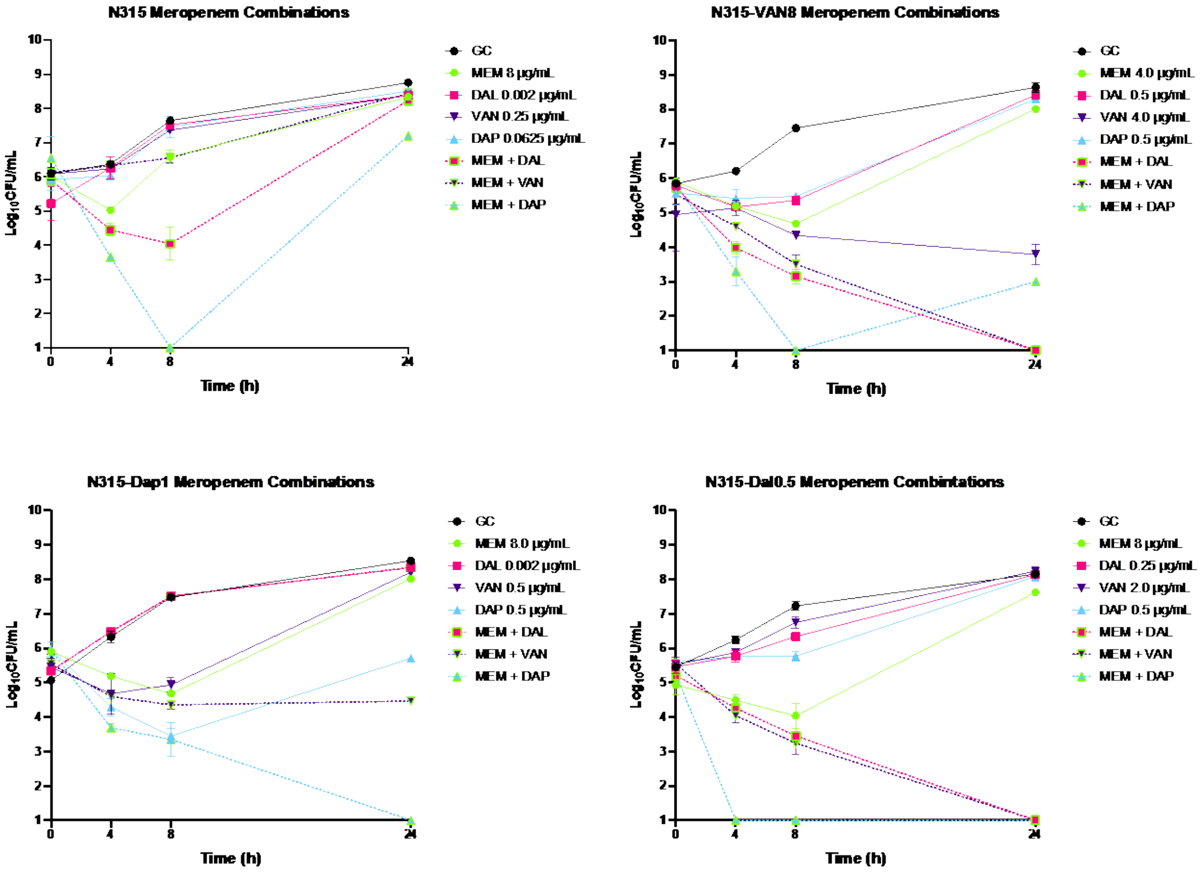

Figure S3. Time kill synergy studies illustrating survival of each N315 strain over 24 hours with exposure to subinhibitory concentrations of ceftriaxone (CRO), dalbavancin (DAL), vancomycin (VAN), daptomycin (DAP) alone and in combination or drug free growth controls (GC).

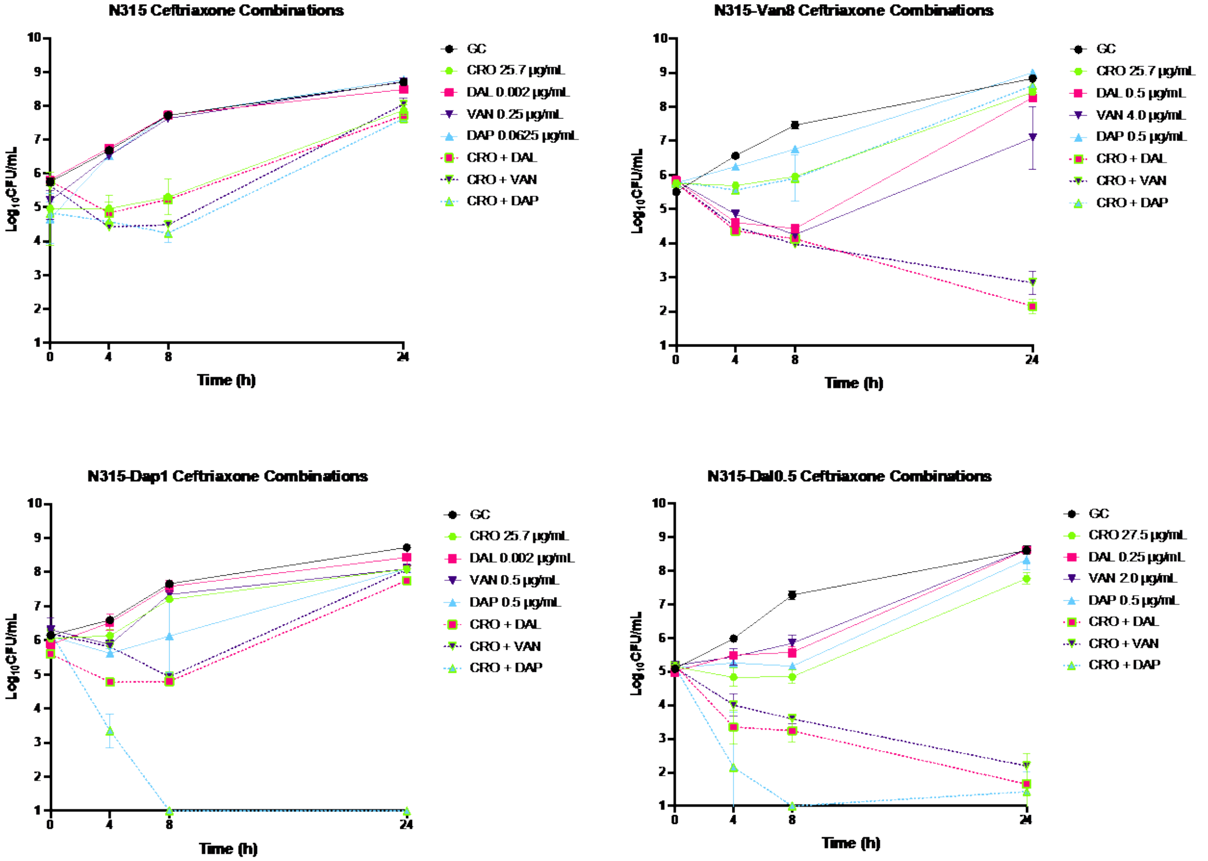

Figure S4. Time kill synergy studies illustrating survival of each N315 strain over 24 hours with exposure to subinhibitory concentrations of cephalexin (LEX), dalbavancin (DAL), vancomycin (VAN), daptomycin (DAP) alone and in combination or drug free growth controls (GC).

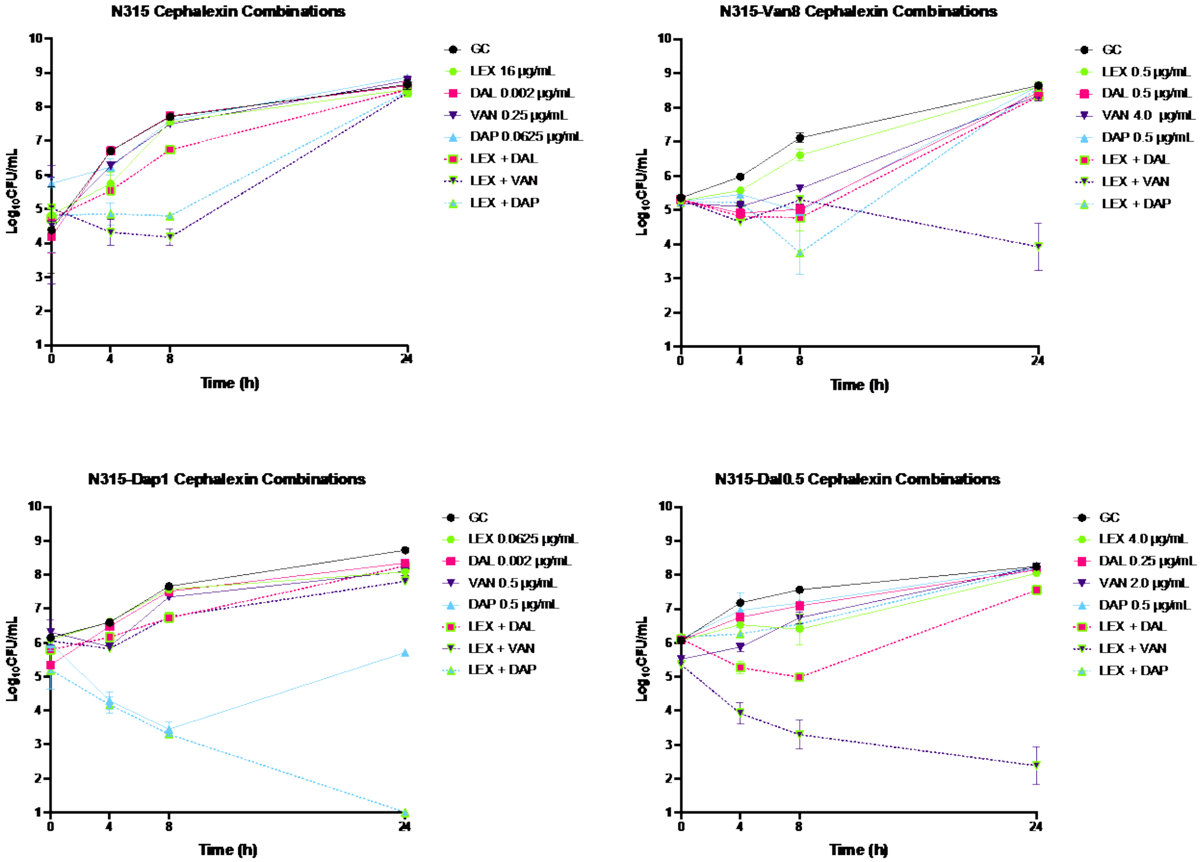

Figure S5. Time kill synergy studies illustrating survival of each N315 strain over 24 hours with exposure to subinhibitory concentrations of cefoxitin (FOX), dalbavancin (DAL), vancomycin (VAN), daptomycin (DAP) alone and in combination or drug free growth controls (GC).

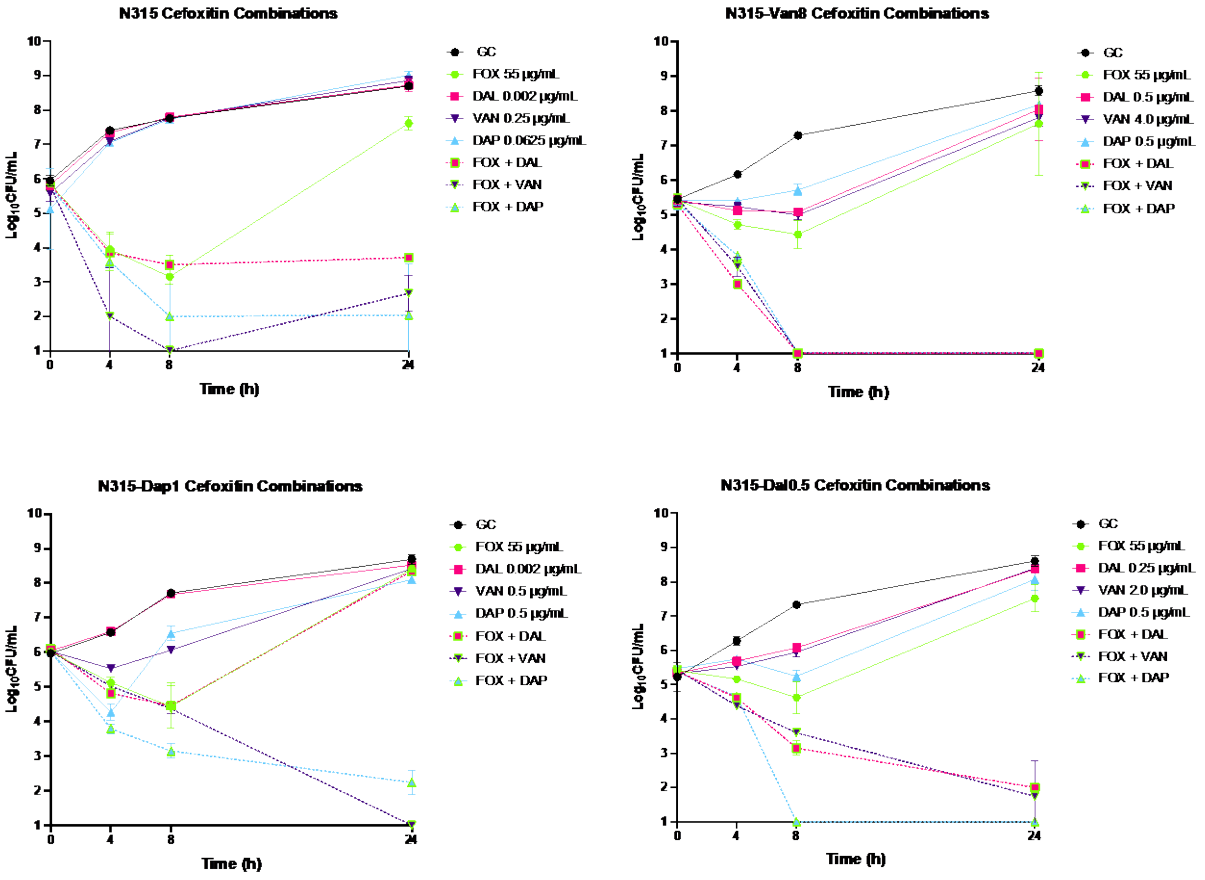

Figure S6. Time kill synergy studies illustrating survival of each N315 strain over 24 hours with exposure to subinhibitory concentrations of ceftaroline (CPT), dalbavancin (DAL), vancomycin (VAN), daptomycin (DAP) alone and in combination or drug free growth controls (GC).

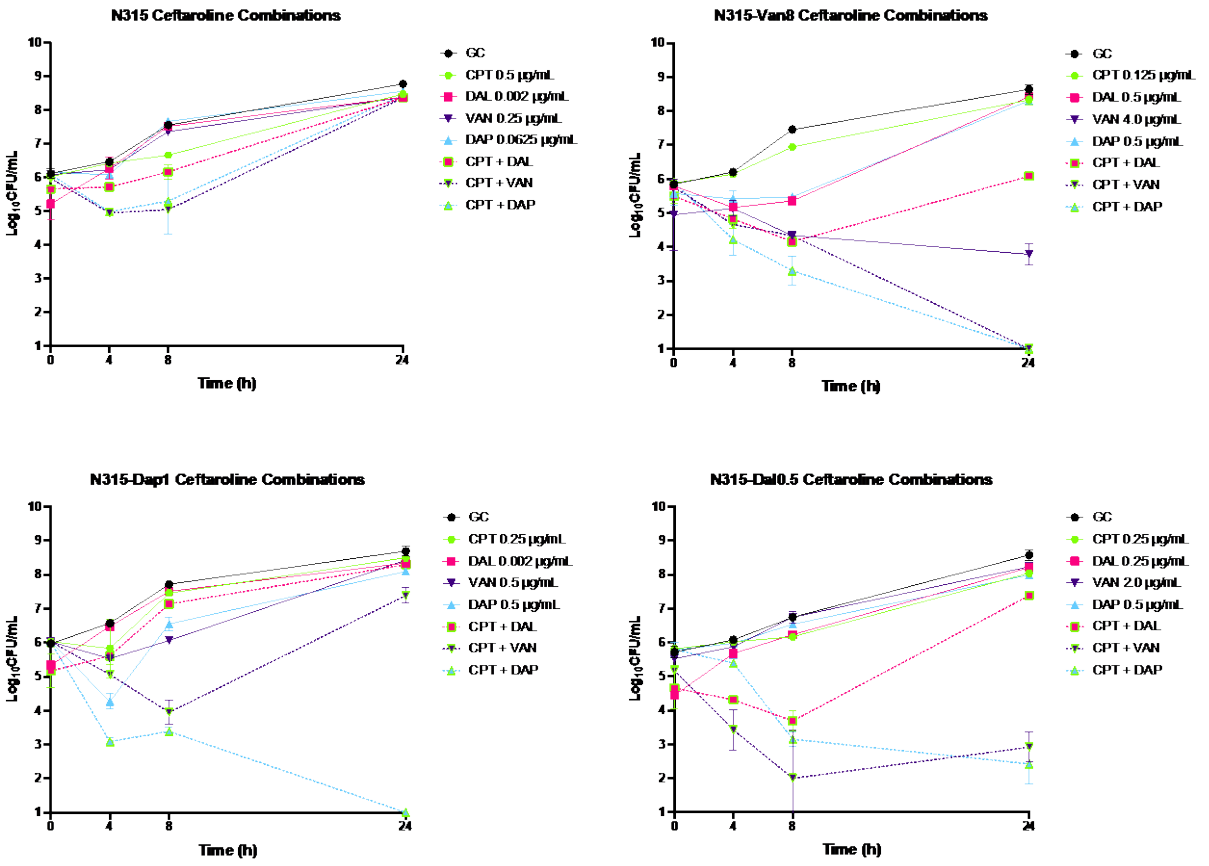

Figure S7. The scores plot of N315 and N315-derived strains obtained by PCA analysis based on lipidomic data acquired in negative mode. Samples in a dash circle indicated that they have similar lipid profiles.

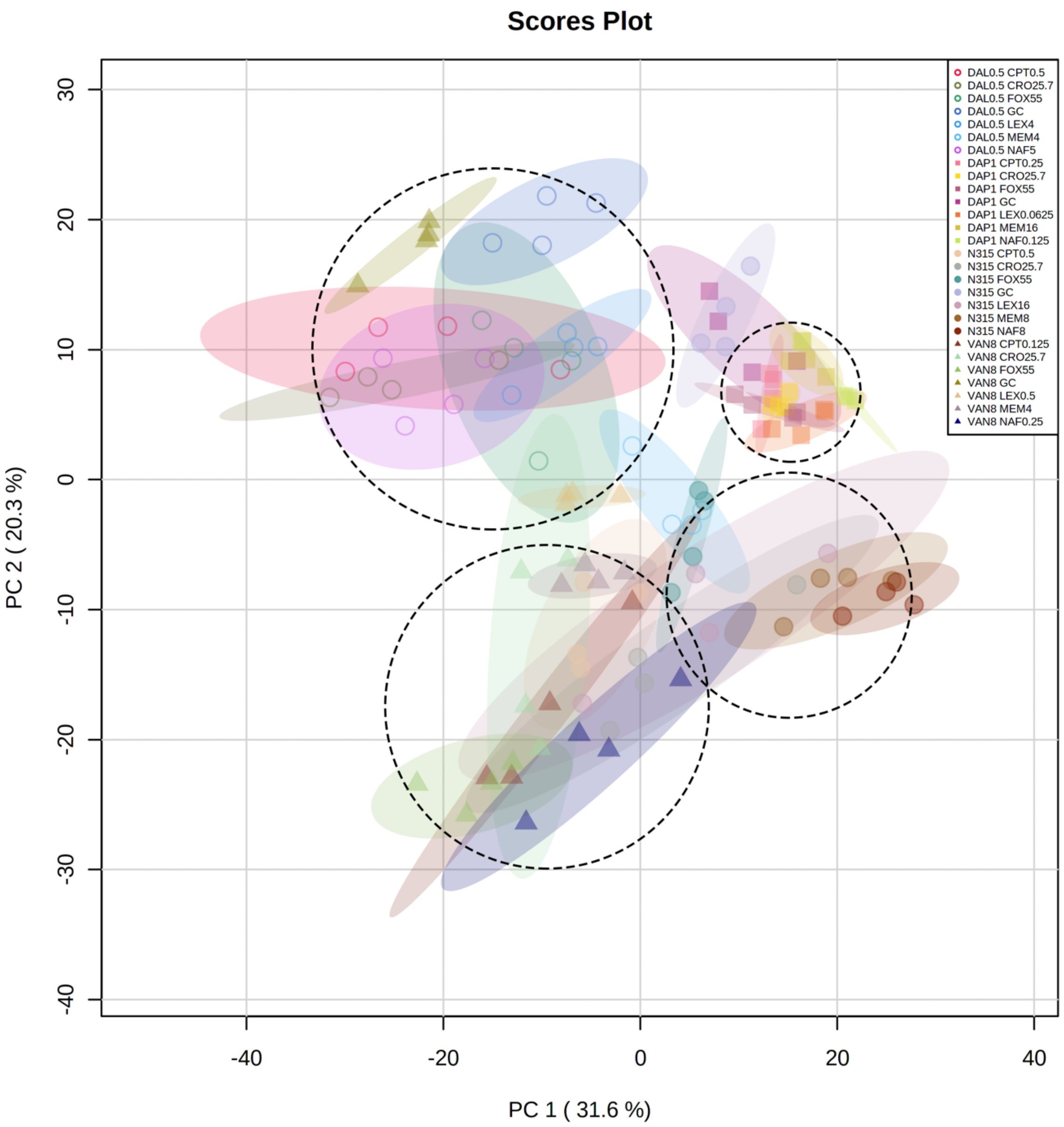

Figure S8. The abundance of lysylPGs in N315-DAP1 strains treated with various beta-lactams. Data were normalized by the sum of peak areas of all compounds.

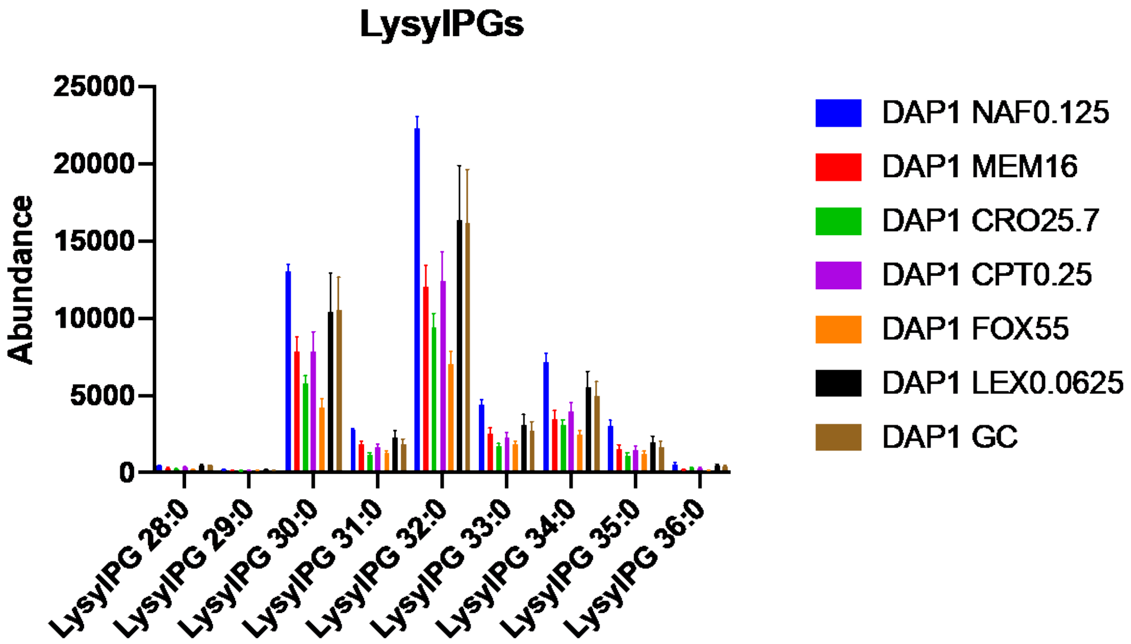

Figure S9. The abundance of cardiolipins in N315-DAP1 strains treated with various beta-lactams. Data were normalized by the sum of peak areas of all compounds.

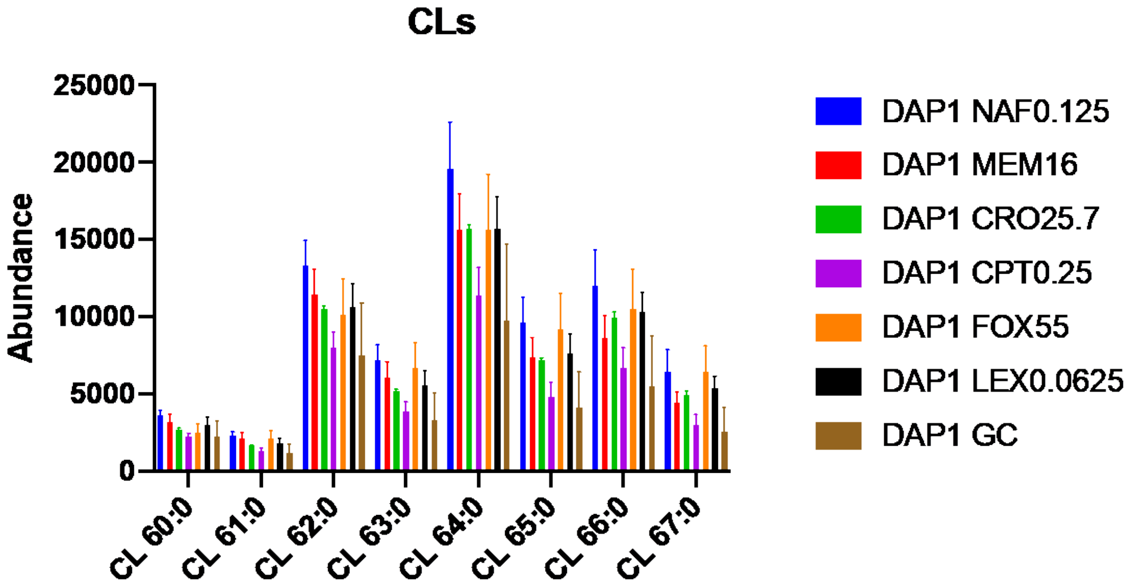

Figure S10. The abundance of cardiolipins in N315-VAN8 strains treated with various beta-lactams. Data were normalized by the sum of peak areas of all compounds.

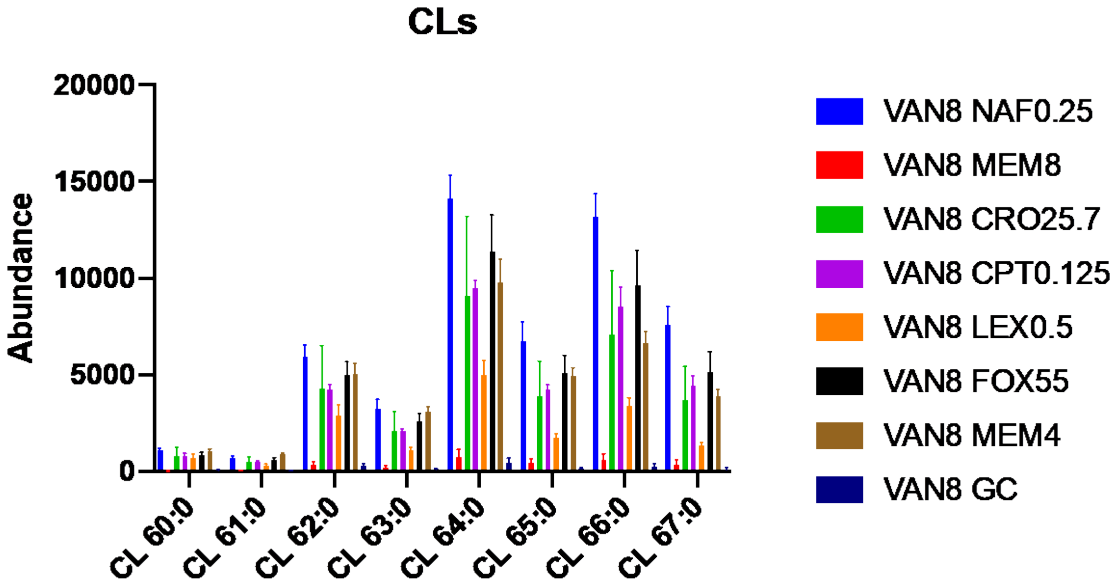

Figure S11. The abundance of cardiolipins in N315-DAL0.5 strains treated with various beta-lactams. Data were normalized by the sum of peak areas of all compounds.

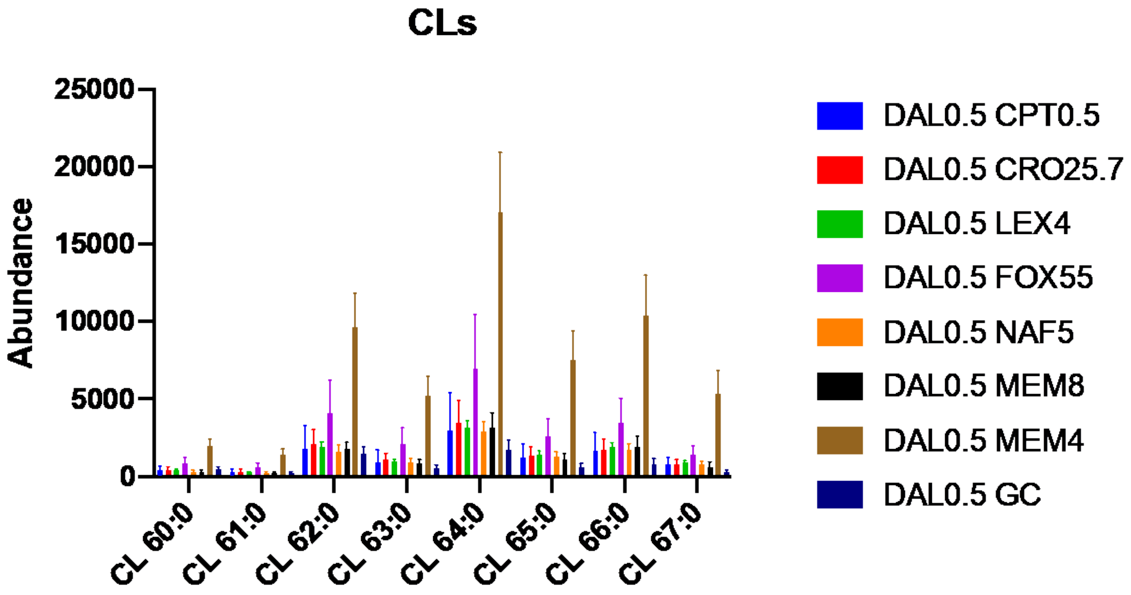

Figure S12. The abundance of cardiolipins in N315 strains treated with various beta-lactams. Data were normalized by the sum of peak areas of all compounds.

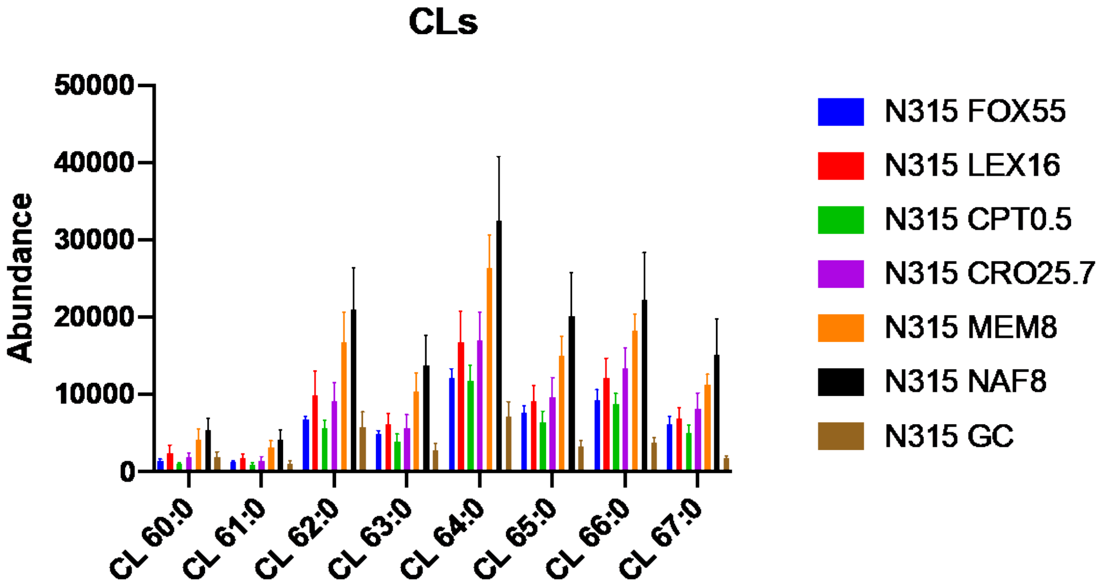

Figure S13. The correlation between the ratio of PGs/LPGs and DAP-beta-lactam measures of synergy by combination MIC (Left panel; 1/fold change in MIC in the presence of subinhibitory beta-lactams) by time kill (right panel; change in log_{10}CFU/mL).

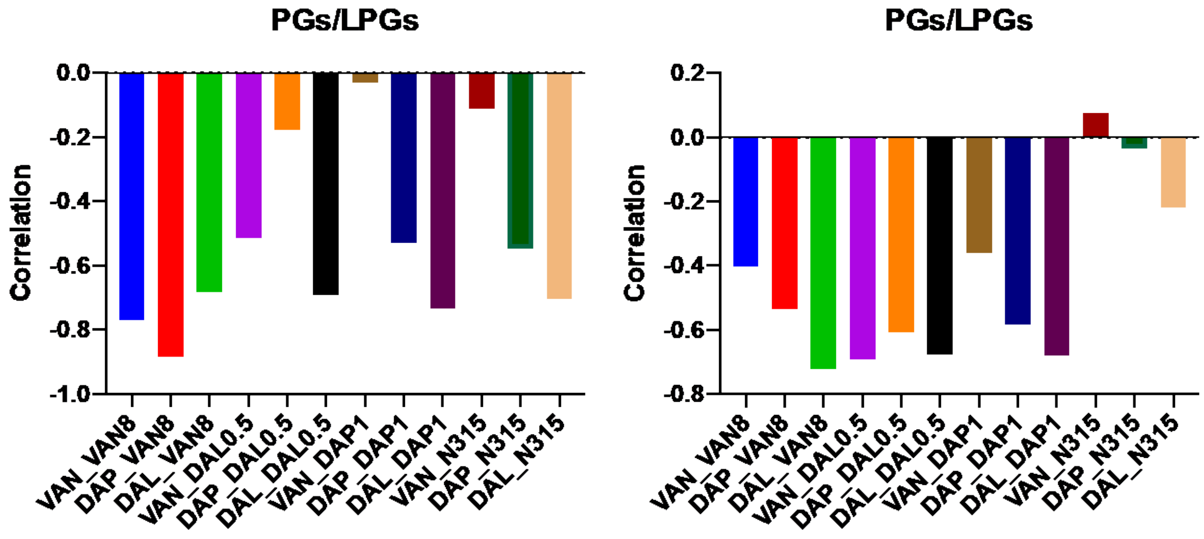

Figure S14. Fluorescence polarities of N315 strains treated with or without beta-lactams. Notably, the lower polarity value represents a higher membrane fluidity, and vice versa. GC represented growth control without beta-lactams.

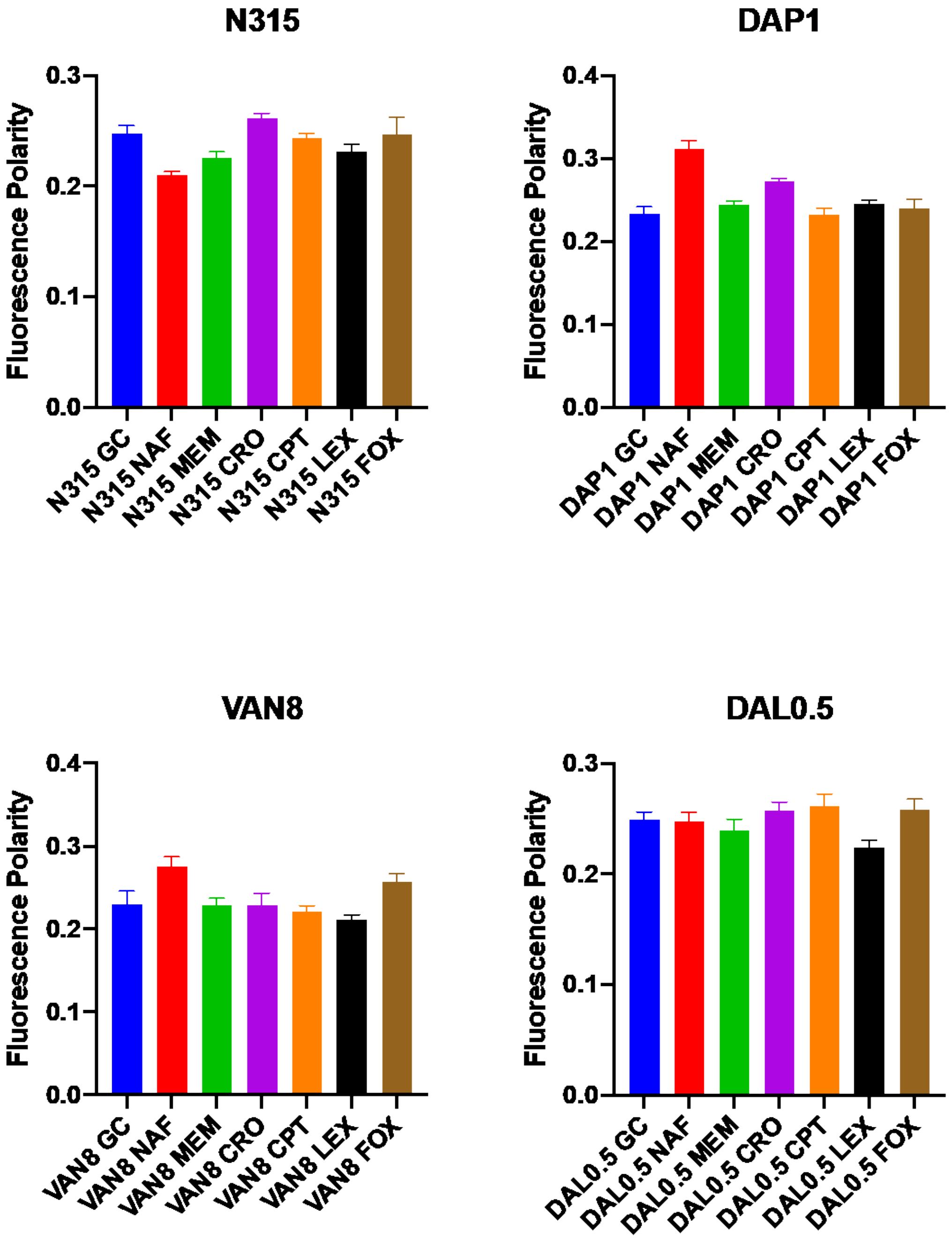

Figure S15. The correlation between membrane fluidity (fluorescence polarity; FP) and measures of synergy by time kill (change in log_{10}CFU/mL) or by combination MIC (1/fold change in MIC in the presence of subinhibitory beta-lactams).

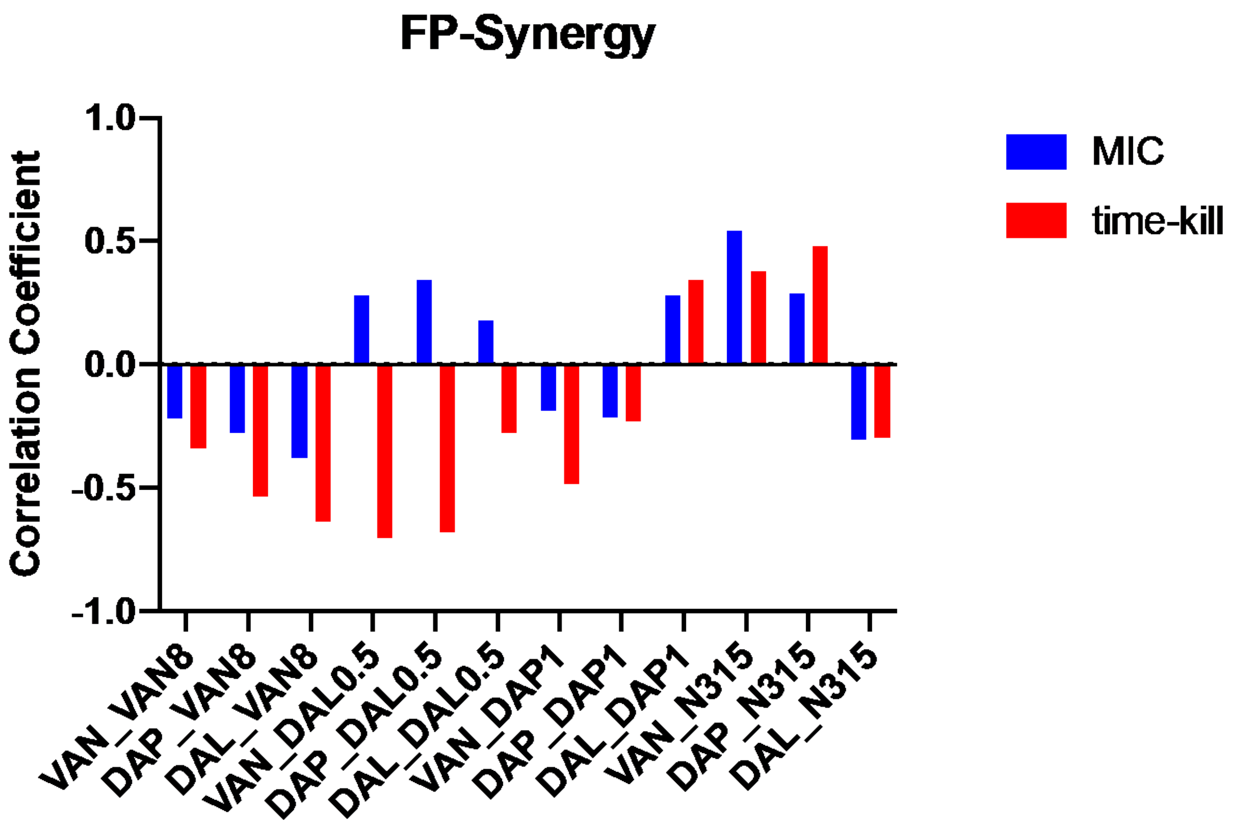

Table S1. Identification of main lipids in N315 and N315-derived strains.

Putative ID	RT (min)	m/z (obs.)	m/z (theo.)	Adduct	Accuracy (ppm)	CCS^{ref} ($Å^2$)[1-3]	CCS^{obs} ($Å^2$)	RSD (%)
CL 60:0	4.46	647.4465	647.4475	[M-2H]2-	-1.50	--	199.8	--
CL 61:0	4.50	654.4545	654.4553	[M-2H]2-	-1.27	--	199.7	--
CL 62:0	4.50	661.4623	661.4632	[M-2H]2-	-1.37	--	201.5	--
CL 63:0	4.50	668.4700	668.4710	[M-2H]2-	-1.43	--	203.2	--
CL 64:0	4.50	675.4781	675.4788	[M-2H]2-	-0.98	--	203.1	--
CL 65:0	4.50	682.4854	682.4866	[M-2H]2-	-1.73	--	204.8	--
CL 66:0	4.50	689.4934	689.4945	[M-2H]2-	-1.59	--	206.5	--
CL 67:0	4.50	696.5012	696.5023	[M-2H]2-	-1.54	--	206.5	--
DGDG 29:0	1.02	868.5994	868.5992	[M+NH4]+	0.28	298.3	293.4	-1.64
DGDG 30:0	1.02	882.6159	882.6148	[M+NH4]+	1.28	302.4	297.2	-1.73
DGDG 31:0	0.98	896.6312	896.6305	[M+NH4]+	0.77	305.4	300.9	-1.47
DGDG 32:0	0.98	910.6479	910.6461	[M+NH4]+	1.93	308.5	304.6	-1.25
DGDG 33:0	0.98	924.6627	924.6618	[M+NH4]+	0.99	311.5	309.6	-0.61
DGDG 34:0	0.93	938.6785	938.6774	[M+NH4]+	1.18	311.8	312.0	0.07
DGDG 35:0	0.93	952.6943	952.6931	[M+NH4]+	1.29	317.1	314.4	-0.84
DGDG 36:0	0.93	966.7094	966.7087	[M+NH4]+	0.70	326.8	318.1	-2.67
DGDG 37:0	0.90	980.7230	980.7244	[M+NH4]+	-1.46	--	320.5	--

LysylPG 28:0	7.44	795.5491	795.5494	[M+H]+	-0.36	284.7	285.5	0.27
LysylPG 29:0	7.41	809.5640	809.5650	[M+H]+	-1.29	287.8	284.6	-1.10
LysylPG 30:0	7.37	823.5812	823.5807	[M+H]+	0.62	290.4	287.2	-1.11
LysylPG 31:0	7.34	837.5960	837.5963	[M+H]+	-0.34	293.9	289.7	-1.43
LysylPG 32:0	7.34	851.6127	851.6120	[M+H]+	0.82	296.8	293.5	-1.11
LysylPG 33:0	7.29	865.6275	865.6276	[M+H]+	-0.07	299.8	297.3	-0.85
LysylPG 34:0	7.29	879.6439	879.6433	[M+H]+	0.66	302.5	299.7	-0.91
LysylPG 35:0	7.25	893.6593	893.6589	[M+H]+	0.48	305.4	302.2	-1.05
LysylPG 36:0	7.25	907.6746	907.6746	[M+H]+	0.05	308.7	304.7	-1.31
PG 29:0	2.10	679.4544	679.4555	[M-H]-	-1.64	255.2	254.1	-0.42
PG 30:0	2.07	693.4707	693.4712	[M-H]-	-0.69	258.7	260.2	0.59
PG 31:0	2.02	707.4854	707.4868	[M-H]-	-1.96	262	264.7	1.03
PG 32:0	1.98	721.5024	721.5025	[M-H]-	-0.12	265.1	269.1	1.53
PG 33:0	1.91	735.5169	735.5181	[M-H]-	-1.65	268.6	273.5	1.84
PG 34:0	1.86	749.5330	749.5338	[M-H]-	-1.02	271.6	276.4	1.78
PG 35:0	1.79	763.5482	763.5494	[M-H]-	-1.52	275	279.3	1.56
PG 36:0	1.79	777.5631	777.5651	[M-H]-	-2.57	277.9	283.6	2.06
PG 37:0	1.76	791.5787	791.5807	[M-H]-	-2.49	282.8	286.4	1.29
PG p30:1	1.36	675.4604	675.4607	[M-H]-	-0.39	255.3	255.7	0.16

PG p31:1	1.36	689.4752	689.4762	[M-H]-	-1.50	258.5	258.7	0.08
PG p32:1	1.36	703.4913	703.4919	[M-H]-	-0.89	262.1	263.2	0.42
FFA 14:0	0.78	227.2010	227.2016	[M-H]-	-2.74	166.3	166.6	0.17
FFA 15:0	0.78	241.2165	241.2173	[M-H]-	-3.19	169.1	168.1	-0.59
FFA 16:0	0.74	255.2333	255.2329	[M-H]-	1.55	171.9	173.7	1.03
FFA 17:0	0.74	269.2477	269.2486	[M-H]-	-3.46	174.7	177.2	1.42
FFA 18:0	0.71	283.2642	283.2642	[M-H]-	-0.02	179.1	180.7	0.87
FFA 19:0	0.71	297.2789	297.2799	[M-H]-	-3.41	181.9	184.1	1.23
FFA 20:0	0.67	311.2933	311.2955	[M-H]-	-6.97	186.2	187.6	0.74
FFA 21:0	0.67	325.3102	325.3112	[M-H]-	-2.98	188.9	191.0	1.11
FFA 22:0	0.67	339.3253	339.3268	[M-H]-	-4.40	191.6	194.4	1.46

Table S2. The membrane fluidity (Fluorescence Polarization Index) of N315 strains treated with or without beta-lactams.

**Strain**	**Treatment**	**Fluidity (Polarization Index)**
N315	None (QC)	0.247±0.008
	Nafcillin (NAF)	0.210±0.004
	Meropenem (MEM)	0.226±0.005
	Ceftriaxone (CRO)	0.261±0.004
	Ceftaroline (CPT)	0.243±0.004
	Cephalexin (LEX)	0.231±0.007
	Cefoxitin (FOX)	0.246±0.016
N315-DAP1	None (QC)	0.234±0.008
	Nafcillin (NAF)	0.311±0.010
	Meropenem (MEM)	0.244±0.005
	Ceftriaxone (CRO)	0.272±0.003
	Ceftaroline (CPT)	0.233±0.007
	Cephalexin (LEX)	0.245±0.005
	Cefoxitin (FOX)	0.240±0.011
N315-VAN8	None (QC)	0.230±0.016
	Nafcillin (NAF)	0.275±0.012
	Meropenem (MEM)	0.229±0.008
	Ceftriaxone (CRO)	0.229±0.014
	Ceftaroline (CPT)	0.221±0.007
	Cephalexin (LEX)	0.211±0.007
	Cefoxitin (FOX)	0.256±0.010
N315-DAL0.5	None (QC)	0.249±0.007
	Nafcillin (NAF)	0.247±0.009
	Meropenem (MEM)	0.239±0.011
	Ceftriaxone (CRO)	0.257±0.008
	Ceftaroline (CPT)	0.261±0.011
	Cephalexin (LEX)	0.224±0.006
	Cefoxitin (FOX)	0.258±0.010

References:
1. Hines, K. M.; Waalkes, A.; Penewit, K.; Holmes, E. A.; Salipante, S. J.; Werth, B. J.; Xu, L., Characterization of the Mechanisms of Daptomycin Resistance among Gram-Positive Bacterial Pathogens by Multidimensional Lipidomics. *mSphere* **2017,** *2* (6), e00492-17.
2. Hines, K. M.; Xu, L., Lipidomic consequences of phospholipid synthesis defects in Escherichia coli revealed by HILIC-ion mobility-mass spectrometry. *Chem. Phys. Lipids* **2019,** *219*, 15-22.
3. Ross, D. H.; Cho, J. H.; Zhang, R.; Hines, K. M.; Xu, L., LiPydomics: A Python Package for Comprehensive Prediction of Lipid Collision Cross Sections and Retention Times and Analysis of Ion Mobility-Mass Spectrometry-Based Lipidomics Data. *Anal. Chem.* **2020,** *92*, 14967–14975.
